# Partners in Silencing: Decoding the Mammalian Argonaute Interactome

**DOI:** 10.3390/ncrna11040062

**Published:** 2025-08-19

**Authors:** Srinaath Narasimhan, Stefan J. Erkeland

**Affiliations:** Department of Immunology, Erasmus Medical Centre, Dr. Molenwaterplein 40, 3015 GD Rotterdam, The Netherlands; s.narasimhan@erasmusmc.nl

**Keywords:** miRISC, AGO, RISC cofactors, PTMs, gene silencing

## Abstract

MicroRNAs (miRNAs) are key post-transcriptional regulators controlling gene expression across several cellular processes, including development, proliferation, and apoptosis. Their biogenesis involves a multi-step pathway, including the processing of primary transcripts and the assembly of the RNA-Induced Silencing Complex (RISC) with Argonaute (AGO) proteins at its core. This review provides a comprehensive overview of the molecular dynamics of miRNA-loaded RISC (miRISC), focusing on the post-translational modifications, the interactors of AGOs and the mechanisms that fine-tune and coordinate miRISC activity. The composition of miRISC influences AGO stability, localization, and silencing efficiency, thereby maintaining cellular homeostasis and development and mediating the response to various types of cellular stress. Uncommon regulatory mechanisms, including AGO modifications during, e.g., hypoxia or Type 2 T cell responses and miRISC functionality, with myriad RNA-binding proteins (RBPs), will be discussed. This review aims at highlighting the recent advances in the understanding of the intricate regulation of miRISC-driven gene silencing.

## 1. Introduction

The discovery of the microRNA (miRNA, miR), *lin-4*, by Nobel prize laureates Viktor Ambros and Gary Ruvkun in 1993, revealed that short RNA fragments lacking protein coding ability called miRNAs regulate the expression of protein-coding mRNAs such as *lin-14* in *C. elegans* [1,2]. A few years later, another miRNA, *Let-7*, was discovered regulating developmental programs in *C. elegans* by targeting mRNA transcripts, including *lin-28*, *lin-41*, and *lin-42* [3], fundamentally changing our understanding of gene regulation. Mounting data show that miRNAs are critical pleiotropic post-transcriptional regulators in various cellular processes such as cell proliferation, development, and apoptosis, thereby controlling the expression of a vast majority of genes in mammalian cells. Thousands of miRNAs have been identified in numerous species and are listed in the miRNA database miRbase, which currently contains 1917 human entries [4].

Most miRNAs are well-conserved across species, indicating their importance in controlling gene expression. In general, the biogenesis of a miRNA starts with its expression in the nucleus from either mono- or multi-cistronic miRNA containing sequences by the action of RNA Polymerase II (Pol II) [5]. Only a limited subset of miRNAs downstream of Alu elements may also be transcribed by Pol III [6]. Recent work shows that in case of Pol III-transcribed *miR-106a*, the polymerase activity can be promoted by transcription factor Signal Transducer And Activator Of Transcription 3 (STAT3), indicating that some transcription factors that are typically associated with Pol II can also stimulate Pol III-mediated transcription of specific miRNAs [7].

Approximately 40–60% of all miRNAs reside in intronic regions of protein encoding transcripts or long non-coding RNAs, suggesting co-expression with their host gene [8,9]. However, the expression of 35% of intronic miRNAs show no correlation with their host gene and their transcription is driven by independent promoters, allowing for tissue- and cellular condition-specific regulation independently of the expression of their host gene [10,11]. An example is *MIR139*, which is located in intron-1 of the *PDE2A* host gene. *MIR139* is transcriptionally regulated by two upstream enhancer regions and silenced in acute myeloid leukemia by a POLR2M-mediated mechanism involving paused transcription [12]. The resulting primary miRNA transcript (pri-miR) containing hairpins are normally processed by the RNAse III enzyme DROSHA and the double stranded RNA (dsRNA) binding protein and Microprocessor Complex Subunit DGCR8 into precursor miRNA transcripts (pre-miR) in the nucleus [13]. However, some intronic miRNAs, the so-called mirtrons, are spliced and then processed into miRNAs, bypassing the DROSHA/DGCR8 cleavage step [14].

The pre-miRNAs are exported to the cytoplasm by Exportin-5 (XPO-5), and further processed by the RNAse III enzyme DICER1 into a ~22 nucleotide (nt) miRNA duplex [15]. During this process, two double stranded RNA-Binding Proteins (dsRBPs), namely human immunodeficiency virus-1 transactivating response RNA-binding protein (TRBP) and protein activator of PKR (PACT), interact with DICER1 and recruit AGO to form the RISC-loading complex (RLC) [15,16,17]. The loading of miRNAs into AGO2-RISC is an energy dependent process requiring ATP breakdown by the heat shock cognate 71 KDa protein (HSC70) and heat shock protein 90 (HSP90) [18]. Heat shock cognate 71 KDa protein (HSC70) binds to AGO and structurally opens the protein. Along with HSC70, co-chaperones such as FKBP Prolyl Isomerase 4 (FKBP4) and p23 interact with human AGO2 to facilitate the activities of HSP90 during RISC loading [19]. Within the miRNA duplex, the strand that possesses the least thermodynamic stability at its 5′-end, the so-called guide-strand, is loaded in the final RNA-Induced Silencing Complex (RISC) [13]. The other strand, known as the passenger strand, is removed from the RISC and subsequently degraded. This process is mediated in a slicer-dependent or independent manner by AGO proteins. AGO2, having slicer activity, nicks the passenger strand in the central region while still being part of the miRNA duplex, thereby destabilizing and inducing the ejection of this strand [20]. The full degradation of the passenger strand is executed by the Component 3 Promoter of RISC (C3PO), which is an endonuclease complex that exist of Translin (TSN) and TRanslin Associated factor X (TRAX) [21]. In contrast, non-slicer AGO proteins (AGO1/3/4) cannot nick the passenger strand. Instead, these AGO proteins are able to separate the passenger strand from the guide-strand by exploiting the mismatches within the miRNA duplex. This process is accelerated when the mismatches are present at the nucleotide position 13–16 of the guide strand within the miRNA duplex [22]. The endonuclease complex involved in the degradation of the passenger strand in case of AGO1/3/4 is unknown.

**Figure 1:**
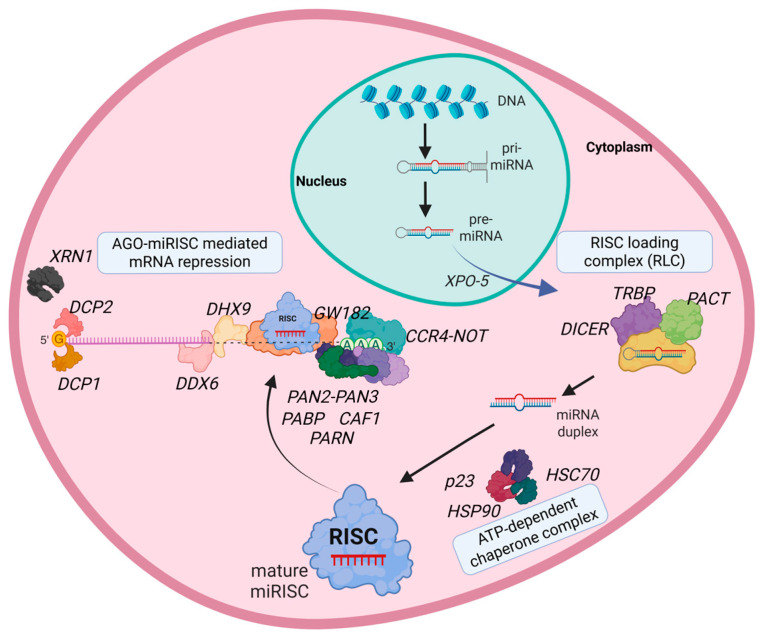
Canonical interactors of AGO involved in miRNA processing. MiRNAs are small non-coding RNAs that are transcribed as independent transcripts or are spliced from introns of protein coding transcripts or non-coding RNAs within the nucleus of cells (green). The pri-miRNA transcript processing factors DROSHA and DGCR8 and XPO-5 that drives the cytoplasmic localization of pre-miRNAs are shown. In the cytoplasm (pink), pre-miRNAs undergo further processing by DICER, along with cofactors PACT and TRBP, forming the RISC RLC. The resulting mature miRNAs (depicted in red) are subsequently loaded into AGOs. This RISC loading process is facilitated by ATP hydrolysis and the action of chaperone proteins such as HSC70, HSP90, and p23. Additional factors involved in miRNA-mediated gene silencing are depicted, including the following. (1) De-adenylation complexes: CCR4-NOT, PAN2-PAN3, PABP, CAF1, and PARN. (2) RNA helicases: DDX6, DHX9; Decapping complex: DCP1/2; 3. 5′ to 3′ exoribonucleases: XRN1. These components collectively contribute to the regulation of mRNA stability and degradation, ensuring precise post-transcriptional gene regulation by miRNAs.

RISC-loaded miRNAs bind to reverse complementary sequence motifs in the 3′-untranslated regions (UTR) of target mRNAs. This miRNA–mRNA interaction is mainly dependent on nucleotides 2 till 8 of the miRNA, the so-called seed [23]. The canonical function of miRNA-loaded RISC is to mediate the cleavage or translational repression of target mRNAs [23,24]. Target mRNA repression mediated by miRISC can be classified into three major mechanisms of action: 1. direct mRNA cleavage by AGO2; 2. de-adenylation-dependent de-capping of mRNA; and 3. de-adenylation-independent mRNA destruction [25]. The first step towards de-adenylation dependent mRNA decay is the binding of AGO2 to the Glycine–tryptophan protein of 182 kDa family of proteins (Trinucleotide repeat-containing gene [TNRC6A, TNRC6B, TNRC6C]) that acts as a binding template for other factors [26]. For example, the Carbon Catabolite Repression 4–Negative On TATA-less (CCR4-NOT) complex, specifically with NOT1 and NOT9, Poly(A) Specific Ribonuclease Subunit 2/3 (PAN2-PAN3), and CCR4-NOT Transcription Complex Subunit 7 (CAF1), are principal de-adenylation factors bound by miRISC [25,26,27]. CCR4-NOT and PAN2-PAN3 complexes trim the Poly-A tail of target mRNAs, where PAN2-PAN3 shortens the poly-A tail, to an initial length of 50–110 nt. The recruitment of CCR4-NOT complex by Poly-A binding protein (PABP) degrades the remaining poly-A tail, destabilizing the target mRNA [25,27,28]. Poly(A)-Specific Ribonuclease (PARN) is another enzyme bound by miRISCs. Xiaokan et al. found that PARN is recruited to the *TP53* mRNA by the action of *miR-125b*-AGO2 RISC, shortening the poly-A tail and thereby reducing its protein expression [29]. In addition to de-adenylation factors, DEAD-Box Helicase 6 (DDX6), a decapping activator, binds to CCR4-NOT complex and recruits decapping enzymes such as Decapping mRNA 1/2 (DCP1-DCP2) [30,31]. The unstable mRNA is then degraded by the 5′-3′ Exoribonuclease 1 (XRN1), while 3′ to 5′ degradation is catalyzed via the RNA exosome pathway [32,33]. The complete list of canonical interactors are listed in Table 1 and indicated in Figure 1. Unexpected or uncommon RISC activities have been described and depend on post-translational modifications, RISC-interacting proteins, and localization of RISC. In this review, we will highlight novel modifications of AGO proteins, miRISC interactors, and activities.

## 2. The Argonaute Family of Proteins

Argonaute proteins are ~97 KDa well-conserved across species and found in most organisms. The mammalian Argonaute family consist of eight *AGO* genes, which can be divided into two Argonaute subfamilies: the *AGO* subfamily, including *AGO1*, *AGO3*, and *AGO4*, all three clustered together on chromosome 1 and *AGO2*, located on chromosome 8, and the PIWI subfamily, including *HIWI* (*PIWIL1*), *HILI* (*PIWIL2*), *HIWI3* (*PIWIL3*), and *HIWI2* (*PIWIL4*), located on chromosomes 12, 11, 22, and 8, respectively. MiRNAs are loaded into AGO proteins that stabilize the miRNA sequence with half-lives ranging from hours up to days, depending on the sequence of the miRNA and cell type [45,46]. Conversely, the stability of AGO proteins is largely controlled by the availability of miRNAs [47]. Most miRNAs are randomly sorted and associate with all AGOs [48,49,50], except for *miR-451*, as *pre-miR-451* is fully processed by AGO2 and the mature *miR-451* is exclusively loaded into AGO2 [48,49,50]. Recently, Kakumani, P.K., et al. found that RNA-binding protein Cold Shock Domain Containing E1 (CSDE1) binds to *pre*-*miR-451* and interacts with AGO2 and PARN to initiate and further assist the trimming of the hairpin, ultimately resulting in mature *miR-451* [51]. These activities are essential for *miR-451* processing and loss of this mechanism leads to block in erythropoiesis [52].

**Figure 2:**
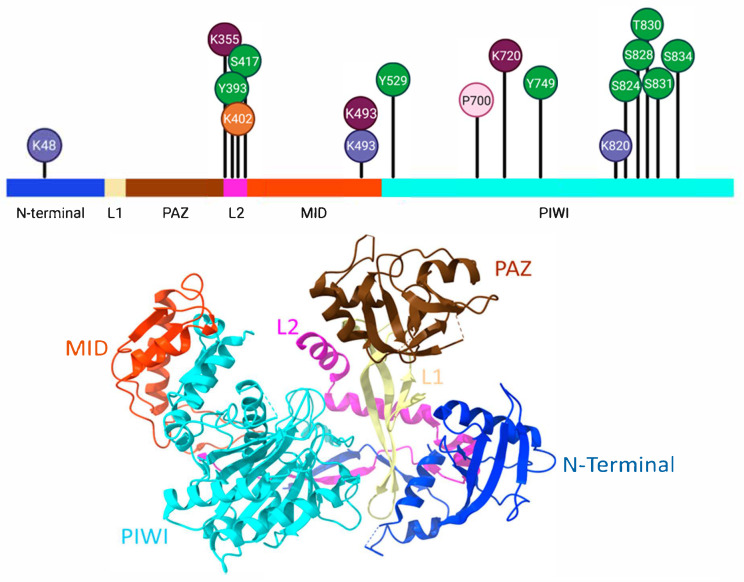
Post-translational modification sites on AGO2. The linear representation of AGO2 protein shows the primary domains as described previously [61]. N-terminal domain (dark blue) serves to unfold dsmiRNA sequences, while the Piwi/Argonaute/Zwille (PAZ) domain (brown) binds to the 3′ end of guide miRNA and immobilizes this sequence within the protein structure. The MID domain (orange) specifically binds the 5′-phosphate of the guide miRNA, and the P-element-induced wimpy testis (PIWI) domain (turquoise) possess the characteristic endonuclease domain of AGO2. The linker L1 (yellow) and L2 (magenta) domains are short peptide sequences that structurally connect the different domains. A 3D representation of AGO2′s different domains (below) is color-matched with the linear scale (top). Several amino acids undergo post-translational modifications such as phosphorylation (green circles), sumolyation (orange circle), ubiquitination (purple circles), acetylation (burgundy circles), and hydroxylation (light pink circle). The position and symbol of the targeted amino acids are given in the circles.

Other studies report that a subset of miRNAs have a bias toward a particular AGO by still-unknown mechanisms [53,54]. The functions of human AGOs are largely considered redundant. However, there are also distinct activities seen that are dependent on variations in post-translational modifications and specific functional domains. For instance, while the knockout of AGO2 is embryonically lethal, mice with genomic deletion of AGO1, AGO3, AGO4, and even the combined knockout of these three AGOs are still viable [55,56]. In humans, only AGO2 has endonuclease activity and slices highly complementary target RNA [57,58]. The fact that AGO2 and AGO3 share the same catalytic tetrad (Asp-Glu-Asp-His), a domain that is lacking in AGO1 and AGO4, suggests that AGO3 may only have slicer activity under specific conditions. Indeed, some miRNAs, including *miR-20a* and tiny RNAs (~18 nt RNAs) activate the slicer activity of AGO3 [58,59]. These RNAs that induce nuclease activity in AGO3 are called cleavage-inducing tiny RNAs (cityRNAs) [60]. AGO proteins are the key factors of RISC, and their expression and functions are strongly regulated by various mechanisms, including post-translational modifications and specific protein interactions. Some of these modifications are discussed below and indicated in Figure 2 and listed in Table 2.

### 2.1. Sumoylation of AGO

Sumo groups (Small Ubiquitin-like Modifiers) play a key role in the activity of miRNA-RISC. For instance, RAN binding protein 2 (RANBP2, NUP358), a GTP-binding protein and a main component of the cytoplasmic filaments of the nuclear pore complex, interacts with E2 enzyme UBC9 and enhances the sumoylation of AGO1 and AGO2 [62,63,64]. Sumoylation is a reversible process where SUMO proteins are attached to target proteins, influencing their activity, localization, and interactions with other proteins. AGO2 interacts with the SUMO E2-conjugating enzyme UBC9,via PINIT domain of SUMO E3 PIAS3 and the E3 ligase RanBP2 and is sumoylated by both SUMO1 and SUMO2, primarily at K402 [63,64]. Sumoylation may have various effects on AGO2 functions. For instance, K402 located in the L2g1 sequence linking the PAZ and PIWI domain, negatively regulates its stability when sumoylated [63]. Earlier findings show that a K402 mutant of AGO2 has similar RNAi knockdown efficiencies compared with *wild-type* AGO2. However, an AGO2 mutant with mutations on the adjacent sites surrounding K402 (^401^VKDE to ^401^AKAA), which cannot interact with UBC9, displayed a clear reduction in RNAi activity as observed in reporter assays [64]. The sumoylation of AGO2 is also required for the interaction of AGO2 with mRNA and enhances the silencing activity of miRNA-RISC on their targets [62]. Interestingly, the sumoylation of AGO and the interaction with mRNA may occur already in the nucleus before it is transported into the cytoplasm [62]. In agreement, RANBP2 interacts with AGO and GW182 proteins and promote the association of target mRNA with miRISC in the nuclear pore complex [65]. Together, these data show that the sumoylation of AGO is critical for the silencing activity of miRISC and promotes AGO2 turnover.

### 2.2. Phosphorylation of AGO

Eukaryotic AGO proteins contain a cluster of residues (amino acids 820-83) called the EI (eukaryotic insertion), which is absent in most prokaryotic AGOs and human PIWIs [66]. The EI insertion contains five highly conserved potential serine phosphorylation sites on position S824, S828, S831, S834, and a Threonine (T) 830, which is only conserved in AGO2 across species [67]. When miRISC binds to a target mRNA, this event promotes the Casein Kinase 1 alpha (CSNK1A1)-mediated phosphorylation of the EI insertion in AGO [66,68]. The CK1α-mediated phosphorylation of AGO largely depends on the stability of the miRNA-mRNA interaction, with 14 nt being the smallest mRNA target length to achieve high stability of the complex. The phosphorylation of AGO is induced with increasing lengths of mRNA targets [68]. However, the complementarity of the mRNA target sequence to the miRNA seed region alone and full miRNA complementary sites does not result in robust phosphorylation [68]. In addition, targets with extended 3′ complementarity (miRNA binding positions nts 12-22), resembling target-directed miRNA decay, triggers significantly less phosphorylation. Only seed target sites (miRNA binding positions nts 2-8) plus supplementary middle region pairing (miRNA binding positions nts 12-17) serves as a trigger for AGO phosphorylation [68]. After miRNA-RISC binding to target mRNA, the phosphorylation of S824-S834 potently inhibits AGO2 interaction with target mRNA. The dephosphorylation is executed by phosphatase PP6/ANKRD52 and reactivates the miRNA-loaded RISC to bind to target mRNA, resulting in a cycle of global miRNA-mediated repression [66,68]. Mutational studies where the S828 residue, but not S824 or T830, was exchanged for an Alanine, showed an overall reduction in the phosphorylation of various sites of AGO2 [69]. Therefore, the phosphorylation of S828 residue is considered to be the driving event that directs the phosphorylation of AGO2 on other sites on the protein, either by CSNK1A1 or by other still unknown kinases [69].

In Non-Small Cell Lung Carcinoma (NSCLC) patients, high levels of AGO2 phosphorylation at S417 (pS417-AGO2), which is surface exposed but not positioned in close proximity of the RNA-binding channel, correlates with a decreased prognosis. One of the drivers may be the increased formation of oncogenic miRISC, which is activated by specific kinase activity [70]. For example, Tank-binding kinase 1 (TBK1) directly binds to AGO2 and phosphorylates S417, thereby stimulating the formation of pS417-AGO2-*miR-21*-RISC, a potent oncogenic driver of NSCLC [70]. S417 phosphorylation affects the selection of miRNAs loaded into AGO2-miRISC and stimulates the formation and activity of miRISC on its targets by unknown mechanisms [70]. Especially, highly expressed oncogenic driver miRNAs such as *miR-21-5p* among others, have a stronger affinity for pS417-AGO2 compared to the non-phosphorylated AGO2 [70]. *miR-21* is an established oncogenic miRNA exerting its suppressive activities on key tumor suppressive targets such as StAR Related Lipid Transfer Domain Containing 13 (STARD13) and Zinc Finger Protein 132 (ZNF132) as implicated in breast cancer [71]. Similarly, Phosphatase and Tensin Homolog (PTEN) and Programmed Cell Death 4 (PDCD4) are direct targets of *miR-21*, evidenced to be downregulated, and support the cancer phenotype in NSCLC [72]. In agreement, TBK1 inhibitor Amlexanox reduced the formation of oncogenic miRISC and is a potential therapeutic approach for the treatment of NSCLC [70].

The highly conserved tyrosine Y529 is located in the small RNA 5′-end-binding pocket, the so-called MID domain, of AGO proteins and can be phosphorylated as well [73]. The mutation of Y529 into E529 (Y529E), introducing a negatively charged amino acid mimicking a phosphorylated tyrosine, impairs miRISC interactions with target mRNAs, and interferes with processing (p) p-body localization and the slicer activity of AGO2 [73]. However phosphorylated Y529 (pY529) is found at low levels in mammalian cells. It is speculated that Y529 phosphorylation occurs only under very specific circumstances to inactivate miRISC-mediated silencing and to induce AGO turnover [67]. Subsequent studies contextualized the role of Y529 phosphorylation under specific cellular conditions. For instance, Y529 phosphorylation alleviates the miRNA-mediated silencing of mRNA encoding pro-inflammatory cytokines in macrophages upon lipopolysaccharide (LPS) stimulation [74]. This causes unloading of miRISC due to reduced miRNA-binding capacity of pY529-AGO [74]. PY529-AGO2 in macrophages is at least in part mediated by p38 mitogen-activated protein kinases (MAPK) [74]. In oligodendrocytes, Y529-AGO2 can also be phosphorylated by FYN, another Src family of non-receptor tyrosine kinases, mediating the translation of the mRNA encoding Myelin basic protein (MBP) [75]. The epidermal growth factor receptor (EGFR) binds to and phosphorylates Y393-AGO2 in response to hypoxia, reducing the interaction between AGO2 and DICER and inhibiting the processing of tumor-suppressing miRNAs [76]. C-Src, a non-receptor tyrosine kinase, binds to AGO and phosphorylate the protein at Y-393, Y-529, and Y-749 [77]. Also, in this study the investigators showed that phosphorylation of AGO2 at Y-393, but not at Y-529 and Y-749, reduces the interaction with DICER and processing of pre-miRNAs and promotes tumorigenesis [77]. Other investigators showed evidence that AGO2 pY529 is as a regulatory switch that inhibits small RNA binding to AGO2. This contributes to oncogenesis by promoting tumor cell growth [77]. The PY393 of AGO is a substrate for protein tyrosine phosphatase 1B (PTP1B), which is targeted by reactive oxygen species (ROS) downstream of H-RAS^V12^ oncogene, resulting in the hyperphosphorylation of AGO2, reduced miRNA loading, and the induction of senescence [78].

E-cadherins interact with AGO2 and mediate the phosphorylation of AGO2 by Extracellular-Signal-Regulated Kinase (ERK), although the residue of phosphorylation on AGO2 remains to be identified [79]. The interaction between E-cadherins and pAGO2 augmented the stability of AGO2, preventing its degradation via the lysosomal pathway and enhanced miRISC-induced silencing of target mRNAs [79]. Together, these data show that miRISC activity is highly regulated by phosphorylation and is controlled by multiple kinases and phosphatases.

### 2.3. Ubiquitination of AGO

The ubiquitination of AGO serves as a regulator of stability and function of miRISC. For instance, mouse Lin41 (mLin41) interacts with and mediates the ubiquitylation and turnover of AGO2 [80]. E3 ubiquitin ligase STIP1 homology and U-box-containing protein 1 (STUB1) is another recently discovered mammalian regulator of RNAi that binds to AGO proteins and facilitates their degradation by the formation of K48-linked polyubiquitin chains [81].

Ubiquitination modulates miRNA abundance via a different pathway involving mRNA targets of miRNA-RISC. For instance, a subset of highly complementary mRNA targets can initiate miRNA degradation, a process called Target-Dependent MiRNA Decay (TDMD) [82] for review [83,84]. In this mechanism, the miRNA binds to the mRNA via their seed sequence, while the 3′-end of the miRNA attracts cellular factors including Terminal Nucleotidyltransferases and RNases, involved in directed tailing and trimming (TDTT), respectively. Recently, a Cullin-RING ubiquitin ligase (CRL), containing the substrate adapter ZSWIM8 mediates TDMD in a tailing and trimming-independent manner. Instead, this mechanism directs the proteasomal decay of miRNA-RISC [85,86]. In this process, ZSWIM8 interacts with AGO2 and presumably ubiquitinates the well-conserved K493, a residue that is essential for TDMD [85]. This TDMD mechanism accounts for the turnover of many miRNAs and explains the half-live of most short-lived miRNAs [86].

Another example of ubiquitin-mediated miRNA inhibition is regulated by cellular stress. During hypoxia, there is a reduction in global miRISC activity due to the prevention of mRNA targeting by AGO2-miRISC that is mediated by the ubiquitin pathway [87]. Herein, RING-Type E3 Ubiquitin Transferase HOIL-1 (HOIL-1L), HOIL-1-Interacting Protein (HOIP), and Shank-Associated RH Domain-Interacting Protein (SHARPIN), part of the Linear Ubiquitin chain assembly complex (LUBAC), interact with AGO2 under hypoxic conditions [87]. This interaction results in the coupling of a Met1 linear poly-ubiquitin (M1-Ubi) chain to K820-AGO2, thereby functionally hindering and blocking the overall mRNA decay [87]. OTU Deubiquitinase With Linear Linkage Specificity (OTULIN), is a deubiquitinating enzyme that specifically removes linear (Met1-linked) poly-ubiquitin chains from AGO2, thereby reactivating miRISC-mediated mRNA silencing [87]. Taken together, external stimuli, such as the deprivation of oxygen, can lead to cross-talk between ubiquitin and miRISC regulatory pathways in cells.

### 2.4. Acetylation of AGO

Protein acetylation is a reversible process, occurs mainly at lysine residues and affects protein–protein interactions. AGO2 may be acetylated under certain circumstances by P300/cAMP response element-binding protein (CBP) at three sites K720, K493, and K355, which can be deacetylated by HDAC7 [88]. The acetylation of AGO2 promotes the biogenesis of *miR-19b*, an oncogenic miRNA that is aberrantly upregulated in various types of human cancer [88]. Mutational studies with AGO2 revealed that the two acetylation sites, K493 and K720, but not K355, promotes the biogenesis of *miR-19b*. The selection of pre-*miR-19* for enhanced maturation processing is dependent on the UGUGUG motif in the terminal loop of *pre-miR-19b* and facilitates the assembly of miRISC loading complex [88]. Finally, the investigators revealed that the elevated acetylation of AGO2 promotes the biogenesis of oncogenic *miR-19b* and leads to an aggressive type of lung cancer, as observed in Xenograft mouse models [88].

### 2.5. Poly-ADP-Ribosylation of AGO

Under stress conditions, cells form unique cellular biomolecular condensates that are called stress granules (SG), comprising translation-paused mRNAs, RBPs, non-RBPs, ribosomal proteins, or translation initiation factors. These regions also consist of enzymes that enable Poly (ADP-ribose) protein post-translation modifications on protein targets [89]. AGO proteins (AGO 1-4) localize to SG and are subjected to Poly ADP-ribose catalyzation by Poly ADP-ribose polymerases (PARP) [89]. Poly ADP-ribose inhibits the AGO2-miRISC-mediated mRNA silencing due to steric hinderance or the disruption of electrostatic interactions between the miRNA and mRNA targets [89]. This mechanism blocks miRNA-mediated repression of mRNA targets and saves mRNA transcripts from degradation during stress situations.

### 2.6. Hydroxylation of AGO

Hydroxylation is important for the appropriate folding and stability of proteins. Prolyl 4-hydroxilation is a post-translational modification in which a hydroxyl (−OH) group is added to the fourth position of prolines in a targeted protein. Prolyl 4-hydroxylation of AGO2 is catalyzed by the type I collagen prolyl-4-hydroxylase (C-P4H) complex, specifically at proline 700 (P700), and this modification is crucial for protein stability [90]. The mutation of P700 into an alanine residue reduced the P-body localization of AGO1, AGO2, and AGO4 but not AGO3, while no effects on stress granule localization were observed [90]. In addition, P700 mutations reduced the interaction of AGO with DCP1 and KIAA1093, but maintained the binding to DICER [90]. Together, the hydroxylation of AGO2 is critical for protein stability and effective miRISC-mediated silencing activity.

It is very likely that PTMs act in concert to tune the activities of miRISC. In addition, different PTMs with similar outcomes may be induced by factors activated by different cellular conditions. Combinations of mutated PTMs under various cellular conditions are needed to investigate how PTMs functionally overlap or work cooperatively.

**Table 2:** Post-translational modifiers of AGO proteins.

miRISC Interactors	Interacting AGO	Functionality	Remarks	References
Sumoylation of AGO				
RANBP2	AGO 1-2	Associates with E2 enzyme UBC9 and induces the sumoylation of AGO1/2	-	[62]
PIAS3	AGO2	E3 SUMO protein ligase that catalyzes the final steps of the sumoylation of K402-AGO2	-	[64]
Phosphorylation of AGO				
CSNK1A1	AGO 1-3	Phosphorylation of the EI region of AGO proteins	-	[66,69]
ANKRD52	AGO 2	Dephosphorylation of EI region of AGO2	-	[66]
TBK1	AGO2	Phosphorylates S417-AGO2	Promotes the formation of pS417-AGO2-*miR-21*-RISC in NSCLC	[70]
EGFR	AGO2	Phosphorylates Y103-AGO2	During hypoxia, decreased pY103-AGO2 and DICER interaction	[76]
C-Src	AGO2	Phosphorylates Y393/529/749-AGO2	pY393 sustains interaction with DICER	[77]
PTP1B	AGO2	De-phosphorylates Y393-AGO2	-	[78]
E-cadherins	AGO2	Mediates pAGO2 by ER-kinase signaling	-	[79]
Ubiquitination of AGO				
STUB1	AGO 1-4	Adds polyubiquitin chains to K48-AGO	-	[81]
CRL-ZSWIM8	AGO2	Presumably ubiquitinates K493-AGO2	ZSWIM8 mediates TDMD in a tailing and trimming independent manner	[85]
LUBAC system	AGO2	Couples Met1 linear poly-ubiquitin chain to K820-AGO2	This mechanism occurs mainly during hypoxia	[87]
OTULIN	AGO2	De-linear Deubiquitinating enzyme of AGO2	-	[87]
Acetylation of AGO				
CBP	AGO2	Acetylates K720/K493/K355-AGO2	The acetylation of K493/K720-AGO2 promotes the biogenesis of *pre-miR-19b*	[88]
HDAC7	AGO2	De-acetylate AGO2	-	[88]
Poly ADP-Ribosylation				
PARP	AGO 1-4	Catalyzes Poly (ADP-ribose) polymerization of AGO proteins	Mainly present in stress granules	[89]

## 3. Unexpected Functions of miRISC

Computational analysis predicted that human AGO proteins may have other unexpected functions. For instance, molecular dynamics simulations and structural analyses revealed that human AGO proteins share conformational properties or interact with proteins involved in mitotic phase transitions including, Transitional endoplasmic reticulum ATPase (VCP)53 and DNA polymerase alpha catalytic subunit (POLA1), Tripartite motif-containing protein 67 (TRIM67), Kinesin-like protein (KIF11), and Sine/threonine-protein kinase-1 (PLK1) [91]. However, the experimental validation of the specific activities of AGO during mitosis remains to be performed.

A different form of AGO1 is involved in a mechanism that dampens the miRNA pathway. Interestingly, the amino acid sequence downstream of the canonical stop (TGA) and the downstream in-frame stop codon in the mRNA of AGO1 is highly conserved in mammals, which is suggestive for a functional domain generated by translational read-through [92]. Indeed, the predicted translational read-through results in an additional 33 amino-acid domain, the so-called inter-stop codon region (ISR) at the C-terminus resulting in a protein called AGO1x, which is particularly expressed in brain tissue and to a lesser extend in heart, kidney, and muscle [92]. Unexpectedly, *Let-7a* miRNA binds to a specific motif 10 nucleotide downstream of the canonical stop codon, promotes translational readthrough and increase the expression of AGO1x without altering the canonical translation of the mRNA via the ISR sequence. AGO1x is loaded with miRNAs similar to regular AGO1 and is able to bind to DICER and target mRNA [92]. However, AGO1x-RISC does not repress the translation of targets. This can be largely explained by the inability of AGO1x to interact with GW182, which is an essential component of RISC that mediates the translational repression and degradation of target mRNAs [92]. The overexpression of AGO1x in HeLa cells caused increased global translation and a decreased number of processing (p)-bodies, which are sites for mRNA degradation and translational repression, indicating that a less-popular miRNA-AGO1x-RISC is a global competitor for the activities of canonical miRNA-RISC [92].

### 3.1. Interactors of AGO Proteins That Modulate miRISC Activities

Some AGO interactors are stimulators of RISC activities. For instance, Cold-Shock Domain containing protein (CSDE1) interacts efficiently with AGO2-miRISC via its N-terminal CSD1 domain, whereas the CSD2 domain facilitates mRNA silencing mediated by AGO2-miRISC [93]. The authors found that CSDE1 interacts with P-body assembling protein, MRNA Processing Body Assembly Factor (LSM14A), Decapping MRNA 1 (DCP1)-DCP2 decapping complex and the enhancers of mRNA decapping proteins EDC3 and EDC4, strongly suggesting that CSD1 promotes the decay of miRNA-RISC targeted mRNAs [93].

GW182, also known as trinucleotide Repeat Containing Adapter 6A, TNRC6A) binds to the PIWI domain of all Argonaute proteins through the Glycine–Tryptophan (GW) repeat domain, an interaction that is largely dependent on miRNA [94]. In total, GW182 can bind three different Argonaute proteins that may be loaded with different miRNAs [94]. This finding largely explains the cooperativity in miRNA-mediated gene silencing on binding motifs located within a range of 7–40 bases in 3-UTRs of target genes [95].

Tryptophan, an essential amino acid, controls RISC activity in a very different way. Tryptophan binds directly and specifically to a subset of miRNAs, including *miR-193a-3p, miR-378a-3p*, *miR-29a-3p*, *miR-106a-5p*, and *miR-17-5p*, enhancing the level of mature miRNAs in cells without affecting the levels of precursor miRNA [96]. Additionally, when Tryptophan binds to a miRNA and AGO, this results in a stronger activity on target mRNAs by enhancing AGO2 slicer activity [96]. There is evidence that Tryptophan-bound miRNAs, including *miR-103*, *miR-107*, and *miR-193*, work in concert in the repression of a common set of target genes [96]. Because of the selection of certain miRNAs, thereby regulating common target genes, Tryptophan may function as a molecular switch in biological processes by recruiting multiple miRNAs to target genes. For example, *Caprin-1* mRNA has miRNA binding sites for *miR-103-3p*, *miR-107-3p*, and *miR-193a-3p*. Tryptophan enhances the recruitment of these three miRNAs to the target, thereby enhancing the silencing effect by four-fold [96]. This results in reduced colon cancer metastasis to the liver.

### 3.2. Splicing-Associated Factors

DExD Box Helicase 21, DDX21, is an RNA helicase that interacts with AGO2 indirectly in an RNA-dependent manner in the nucleus of cells, with clear aggregation in the nucleolus [97]. The nuclear expression level of AGO2 largely depends on the expression of DDX21 [97]. AGO2-RNA-DDX21 is involved in the regulation of the alternative splicing of Survival of Motor Neuron 2 (*SMN2*) mRNA in Hela cells [97].

AGO3 has non-canonical functions in the regulation of splicing that are essential for type 2 T cell responses. This activity is controlled by Splicing Factor 3B Subunit 3 (SF3B3), a member of the U2 spliceosome complex, which specifically interacts with AGO3 in the nucleus of Th2 cells and independently from RNA-mediated interactions [98]. AGO3-SF3B3 interactions are required for the regulation of IL-13 expression by controlling global pre-mRNA splicing in T cells, in particular by targeting *Nisch* pre-mRNA to regulate IL-13 expression [98]. Therefore, a non-canonical role of AGO protein in complex with SF3B3 controls Type 2 T cell responses.

### 3.3. mRNA Translation Associated Factors

In neuronal cells, the activities of RISC is regulated by the RNA binding protein Staufen-2 (STAU2), which controls the assembly of ribonucleoprotein involved in the RNA degradation and translational inhibition of RISC targets [99]. STAU2 depletion caused upregulation of the protein expression of key RISC factors, including AGO1, AGO2, DDX6, and DCP1A, without affecting global miRNA expression [99]. In addition, STAU2 knockout cells have an altered AGO1/2 RNP assembly, shifting RISC association from P-bodies to polysomes, thereby repressing the protein expression of miRNA targets [99]. Conversely, the overexpression of STAU2 caused enhanced global translation. These studies show that the intracellular level of STAU2 modulates global translation by controlling RISC activities [99], a mechanism that may play a role in cells other than neurons as well.

In 2014, Phillip J. Kenny et al. identified a unique co-regulatory role of Fragile X Messenger Ribonucleoprotein 1 (FMRP) and RNA helicase Moloney leukemia virus 10 (MOV10) to regulate and protect target mRNAs from miR-mediated translation suppression via AGO2 [100]. Recent data show that FMRP, MOV10, and AGO2 proteins directly interact with each other in this process [101]. MOV10 is involved in miRNA-mediated gene silencing and can exert two different activities on bound target mRNAs: (1) it enhances miRISC function by acting as an RNA helicase that unwinds G-quadruplexes structures in the 3′UTR [101,102], and (2) it protects the bound mRNAs from miRNA-mediated degradation by binding near miRNA recognition elements (MREs) and sterically hindering the accessibility of miRISC [100]. A subset of MOV10-bound mRNAs are positive for FMRP binding sites as well, where MOV10 and FMRP tend to bind in close proximity to MREs [100]. For target mRNAs, where FMRP/MOV10 binding sites do not overlap, MOV10-mediated AGO2 recruitment and subsequent gene silencing was observed. However, mRNAs with FMRP and MOV10 overlapping binding sites, MOV10 is unable to recruit AGO2-miRISC for target suppression [100]. Therefore, FMRP is an important regulatory protein for miRISC activity.

### 3.4. RISC Associated Proteins That Control Localization and miRNA Sorting

A recent study conducted by Lin MC et al. discovered that Caveolin-1 (CAV-1), a plasma membrane protein, directly binds with AGO2 and alters its intracellular localization [103]. The binding of CAV-1 with AGO2 is facilitated by a stretch of aromatic amino acid residues in the CAV-1 binding domain of AGO2. Moreover, within this amino acid stretch, the positive charge of a K212 residue is essential for the interaction, as substituting K212 with a neutral alanine disrupts the binding between CAV-1 and AGO2 [103]. Due to the localization of CAV-1 to the plasma membrane of cells, AGO2 interaction with CAV-1 stimulates target mRNA repression within the plasma membrane pockets. Cancer cell phenotypes such as metastasis and Epithelial-to-Mesenchymal Transition (EMT) are mediated by the simultaneous downregulation of Suppressor of Cancer Cell Invasion (*SCAI*) gene [104,105]. Similarly, AGO2-CAV-1 interaction augments *miR-3613-3p*-mediated suppression of *SCAI* mRNAs in A549 lung cancer cells [103]. In metastatic cancers and carcinomas, CAV1 interaction with AGO2 in the plasma membrane are widely distributed and abundant as compared with primary tumors. CAV-1-AGO2 interactions in the plasma membrane also trigger the sorting and secretion of *miR-3613-3p*-AGO2-RISC in extracellular vesicles (EVs) in the plasma of metastatic cancer patients [103].

Lamin A is another regulator of AGO2 functions. Lamins A and B assemble into structures forming the nuclear lamina, which is important for the exchange of molecules between the nucleus and cytoplasm. Reduced levels of Lamin A results in the transportation of AGO2 into the nucleus of cells where AGO2 binds to nucleoporins, chromatin regulators, RNA-binding protein FAM120A, and RISC targets, thereby inhibiting RISC activity [106]. Lamin A knockout cells have an increased expression of oncogenic miRNAs, including members of the *miR-17~92 cluster*, *miR-21-5p*, and *miR-27a*, caused by an unknown mechanism. The reduced expression or deregulation of Lamin A may be an important event in tumorigenesis, inhibiting miRNA activity or upregulating specific oncogenic miRNAs, thereby reverting the tumor cells to a more primitive state and allowing them to enter into cell cycle and proliferate [106].

### 3.5. Miscellaneous Factors Regulating RISC Activities

There is mounting evidence for uncommon activities of RISC. For instance, a recently discovered well-conserved lipid binding motif within the N-terminal domain of AGO proteins interacts with lipid PI(4,5)P2 and promotes AGOs to condense into phase-separated granules on the endoplasmic reticulum (ER) [107]. Here, AGOs recruit Listerin E3 Ubiquitin Protein Ligase 1 (Ltn1), a conserved E3 ubiquitin ligase to catalyze the ubiquitination of nascent peptides [107]. Next, AGOs act together with the VCP-UFD1-NPL4 (VCP: valosin-containing protein, also known as Ter94; UFD1: Ubiquitin Recognition Factor in ER-Associated Degradation 1; NPL4: Nuclear Protein Localization Protein 4 Homolog) complex to target unwanted proteins for proteasomal degradation [107]. This study provides the first evidence that miRNA-RISC acts in concert with the ribosome quality control machinery to ensure the efficient repression of their unfolded targets.

In breast cancer metastasis, particularly in Triple Negative Breast Cancer (TNBC), LIM, and SH3 Protein 1 (LASP1) interacts with AGO2 in a C-X-C Motif Chemokine Receptor 4 (CXCR4)-dependent manner [108,109]. The direct interaction of LASP1 with AGO2 is facilitated by two key events: the dephosphorylation of S146-LASP1 and the phosphorylation of Y171-LASP1. The binding of LASP1 to AGO2 leads to a specific release of motility and cell-growth related genes, including *CCR7*, *CYCLIND1*, and *eIF4G2* from translational suppression via the inhibition of *Let-7a*-miRISC activity [108]. Due to the CXCL12-mediated activation of CXCR4, LASP1 blocks the binding of AGO2-*Let-7a*-miR-RISCs to target mRNAs. The interaction of LASP1 was also confirmed for AGO1, while its role in miRISC functionality is not known [108]. LASP1 is frequently overexpressed in various types of cancer, including lung, breast, ovarian, and colorectal cancer, and is associated with increased tumor aggressiveness, metastasis, and poor prognosis [110]. As *Let-7a* is a well-known tumor suppressor miRNA [111,112,113], LASP1 may be an interesting and promising candidate for therapeutic targeting for many types of cancer.

LIM Domain Containing 1 (LIMD1) is another miRISC component that binds phosphorylated S387-AGO2 at the L2 linker region via the pre-LIM domains and simultaneously interacts with the N-terminal domain of TNRC6A via its C-terminal LIM domains [114]. The binding of LIND1 with AGO2 is dependent on the phosphorylation of S387-AGO2 by AKT3 kinase [114]. When LIMD1 is knocked out, miRNA silencing shifts from an AGO2-LIMD1-dependent mechanism to a complex involving AGO3 and the LIMD1 family member WTIP [114]. LIMD1 is frequently downregulated or deleted in various types of cancer, particularly in lung and gastric cancers. Therefore, the binding of LIM-domain containing proteins to AGO proteins highlight a phosphorylation-dependent tumor-suppressing mechanism for the post-transcriptional regulation of miRISC target genes in cancers.

**Figure 3:**
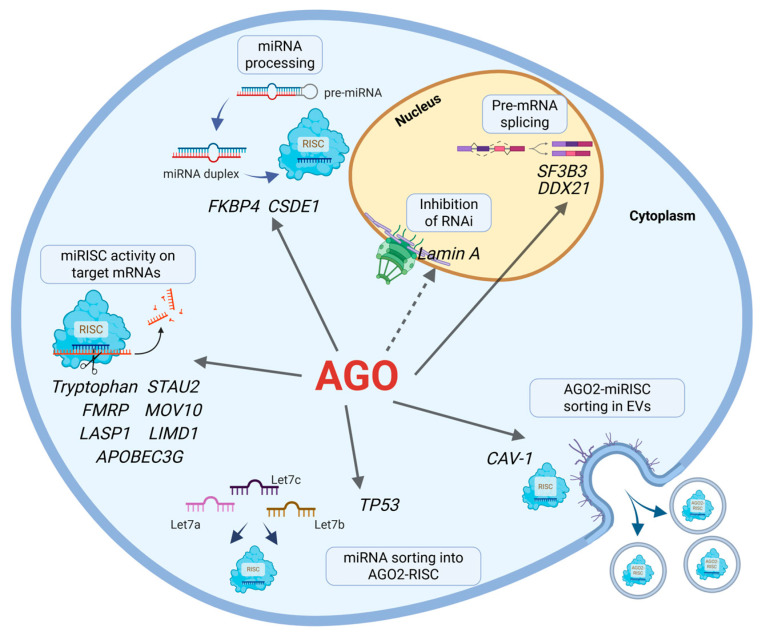
Cofactors of miRISC. Various RBPs or other proteins bind to AGO proteins and modulate or change their functionality in the miRNA-mediated gene silencing pathway. The association of AGO2 with proteins such as STAU2, FMRP, MOV10, LIMD1, or LASP1 controls global transcript levels and/or translation via regulating AGO2-miRISC activity on target mRNAs. MiRNA processing, either at the step of hairpin trimming of pre-miRNA or miRNA loading into RISC, are regulated as a result of AGO2 interaction with FKBP4 or CSDE1. In the nucleus, an indirect consequence of Lamin A downregulation or the heightened nuclear expression of DDX21 alter the localization of AGO2 to the nucleus via unknown mechanisms. Similarly, nuclear AGO3 interacts with SF3B3 spliceosome complex and mediates the splicing of target pre-mRNAs. TP53-associated AGO2 promotes miRNA sorting, especially for *Let-7* family members, into AGO2-miRISC. At the plasma membrane, CAV-1 interacts with AGO2-miR-RISCs, tethering the miRNA-regulatory machinery to the membrane that allows for localized and region-specific mRNA regulation. The dotted line represents an indirect relationship, while solid lines represent direct interaction.

TP53 or p53, the guardian of the genome, is commonly deregulated, mutated, deleted, or silenced in many types of cancers [115]. It has been shown that p53 controls the loading of miRNAs into AGO2 by direct interaction with AGO2 [116]. Upon DNA damage, subsets of miRNAs, including members of the *Let-7* family, are enriched in AGO2, while the expression of these miRNAs remains the same, indicating the active sorting of *Let-7* miRNAs in AGO2-miRISC [116]. Among the *Let-7* family members, only *Let-7d* was not enriched in AGO2-miRISC in a p53-dependent fashion [116]. The authors noticed the enrichment of *Let-7i/c* in AGO2 miRISC in human colorectal carcinoma cells expressing *wild type* p53, but not in DLD1 human colon cancer cells expressing mutant p53 [116]. This miRNA selection could be linked to a change in the conformation of AGO2, favorable for the loading of certain miRNA subsets. The interaction of AGO2 with TP53 in the context of DNA damage response shows that DNA damage can have a widespread impact on the selective regulation of *Let-7* miRNA family and its targets. The novel interactions of AGO as discussed above are listed in Table 3 and are represented in Figure 3.

## 4. Conclusions

This review highlights the multifaceted regulation of AGO proteins and the dynamic composition of miRISC. The interaction of cofactors with AGO-RISCs determines the specificity, efficiency, and context-dependent activity of miRNAs. Post-translational modifications of AGOs results in novel protein–protein interactions, which change miRISC activities. Overall, decoding the AGO-miRISC interactome sheds light on the intricate gene expression regulation under specific cellular conditions. There is mounting evidence for non-canonical miRISC activities [121]. However, the factors involved in these regulatory activities are not fully characterized. Therefore, we expect that there are more molecules that interact with AGOs or other factors that influence miRISC activity to discover. Recent advances in protein capture and sequencing by UV Cross-Linking Immuno-Precipitations (CLIP), enhanced CLIP (eCLIP), and Cross-linking Ligation and Sequencing hybrids (CLASH) have helped us better understand AGO-RNA and RISC–protein interactions [122]. Alongside, high-throughput proteomic approaches have augmented research in the post-translational modifications of AGO and its interactors. Over the years, the field of fluorescent microscopy has expanded with techniques such as Fluorescence resonance energy transfer (FRET), Fluorescence Correlation Spectroscopy (FCS) and super-resolution imaging elevating our knowledge on the dynamics of AGO–protein interaction and RISC assembly in real time [123]. The challenge to overcome in studying miRISC activity is the transient nature of some of these complexes. Techniques such as super-resolution microscopy can be exploited to study the spatiotemporal behavior of RNAs and key components of miRISC and to study miRISC interactions on a single target mRNA [94]. Therefore, understanding these molecular interactions advances our fundamental knowledge of miRISC-mediated gene regulation. In addition, unraveling the miRISC interactome can also help us to develop therapeutics for the treatment of various human diseases including cancer and immunological disorders.

## Figures and Tables

**Table 1:** Canonical interactors of AGO proteins.

miRISC Interactors	AGO Binding	Functionality	Remarks	Refs.
DICER	AGO 1-4	Pre-miRNA processing	-	[5,13,24,34,35]
GW182 family (TNRC6A/B/C)	AGO 1-4	Scaffold protein to recruit other factors of miRISC	-	[36,37]
CCR4-NOT complex	AGO 1-4 via GW182 proteins	Involved in de-adenylation mediated miRISC mechanisms	-	[36,38]
PAN2-PAN3	AGO2	Involved in a 2-stage de-adenylation process with CCR4-NOT complex	-	[27,39]
PABP	AGO 1-4 via GW182 proteins	Involved in de-adenylation mediated miRISC mechanisms	-	[26,40]
CAF1	AGO 1-2	Additional de-adenylase in context-dependent miRISC functionality	-	[26]
PARN	AGO2	Involved in *miR-125b*-AGO2 RISC repression of TP53	Also involved in *pre-miR-451* biogenesis pathway along with AGO2	[29,41]
DDX6	AGO 2	Functions as an activator of decapping enzymes DCP1/DCP2	-	[30]
DHX9	AGO 2-3	RNA helicase that functions in RISC assembly and miRNA loading	-	[42]
DCP1/DCP2	AGO 1-2	Supports de-capping of target mRNAs to promote 5′ to 3′ nucleolytic activity	-	[31]
XRN1	Not directly	Mediates 5′ to 3′ mRNA decay and is recruited by EDC4	Implicated in miRISC-mediated silencing	[43]
TRBP	AGO2 via DICER	Promotes dsRNA binding to RLC	-	[15,44]
PACT	AGO2 via DICER	Promotes dsRNA binding to RLC	-	[15,17]
Hsc70	AGO2	Involved in ATP- dependent conformation change in AGO2 to enable miRNA loading	-	[18]
Hsp90	AGO2		-	[18]
p23	AGO2	Part of the Hsc70/Hsp90 chaperone complex for miRISC loading	-	[19]

**Table 3:** Novel miRISC interactors of AGO proteins.

miRISC Interactors	AGO Binding	Core/ Auxiliary Factors	Functionality	Remarks	References
FKBP4	AGO2	Auxiliary	Promotes ATP-dependent loading of miRNAs in RISC	-	[19]
CSDE1	AGO2	Auxiliary	Binds *pre-miR-451,* AGO2 and PARN to enhance trimming of the hairpin	-	[51,93]
Tryptophan (Amino Acid)	AGO 1-4	Auxiliary	Enhances nuclease activity of AGO2	-	[96]
DDX21	AGO2	Auxiliary	Increases nuclear AGO2 levels	-	[97]
SF3B3 complex	AGO3	Auxiliary	Promotes *Nisch* splicing to increase IL-13 expression in T cells	-	[98]
STAU2	AGO2	Auxiliary	STAU2 regulates global translation by miRISC	-	[99]
FMRP	AGO2	Auxiliary	FMRP-mediates translational control by AGO2-miRISC		[117,118]
MOV10	AGO 1-3	Auxiliary	MOV10 relieves complex secondary structures in 3′-UTRs to augment AGO-miRISC binding to MRE	-	[102,119]
APOBEC3G	Indirect activity	-	Binds MOV10 to inhibit AGO2-miRISC interaction to mRNAs	-	[120]
CAV-1	AGO2	Auxiliary	Sorting of AGO2 RISC into EVs	Observed in breast cancer	[103]
Lamin A	Indirect activity	-	Inhibits RISC activity	-	[106]
PI(4,5)P2	AGO2	-	AGO RNPs condensation on ER membranes	Controls proteasomal degradation of nascent peptides	[107]
LASP1	AGO2	Auxiliary	Enhances translational repression of *Let-7a* targets	This interaction is a CXCR4-dependent mechanism	[108]
LIMD1	AGO2	Auxiliary	A pS387-AGO2-dependent mechanism decreasing translation of mRNA targets	Upon LIMD1 depletion in cells, AGO3-WTIP becomes the dominant miRISC	[114]
TP53	AGO2	Auxiliary	Facilitates sorting of *Let-7* miRNAs into AGO2-miRISC	TP53 may alter the conformation of AGO2 to promote loading of *Let-7* miRNAs into RISC	[116]
FKBP4	AGO2	Auxiliary	Enhances ATP-dependent loading of miRNAs into miRISC		[19]
